# Human VRK1 Is an Early Response Gene and Its Loss Causes a Block in Cell Cycle Progression

**DOI:** 10.1371/journal.pone.0001642

**Published:** 2008-02-20

**Authors:** Alberto Valbuena, Inmaculada López-Sánchez, Pedro A. Lazo

**Affiliations:** Programa de Oncología Translacional, Instituto de Biología Molecular y Celular del Cáncer, Centro de Investigación del Cáncer (CIC), Consejo Superior de Investigaciones Científicas (CSIC), Universidad de Salamanca, Salamanca, Spain; Texas Tech University Health Sciences Center, United States of America

## Abstract

**Background:**

In mammalian cells regulatory proteins controlling the cell cycle are necessary due to the requirements of living in a heterogeneous environment of cell-interactions and growth factors. VRK1 is a novel serine-threonine kinase that phosphorylates several transcription factors and is associated with proliferation phenotypes.

**Methodology/Principal Findings:**

In this report VRK1 has been identified as regulated in the cell cycle. *VRK1* gene expression is activated by the addition of serum to starved cells, indicating it is required for the exit of G0 phase and entry in G1; a response that parallels the re-expression of *MYC*, *FOS* and *CCND1* (cyclin D1) genes, suggesting that *VRK1* is an early-response gene. *VRK1* gene expression is also shutdown by serum withdrawal. The human *VRK1* gene promoter cloned in a luciferase reporter responds similarly to serum. In response to serum, the level of VRK1 protein expression has a positive correlation with cell proliferation markers such as phosphorylated-Rb or PCNA, and is inversely correlated with cell cycle inhibitors such as p27. The elimination of VRK1 by siRNA results in a G1 block in cell division, and in loss of phosphorylated-Rb, cyclin D1, and other proliferation markers. Elimination of VRK1 by siRNA induces a reduction of cell proliferation. VRK1 colocalizes with p63 in proliferating areas of squamous epithelium, and identifies a subpopulation in the basal layer.

**Conclusions/Significance:**

VRK1 is an immediate early response gene required for entry in G1, and due to its implication in normal cell proliferation and division, might be a new target for development of inhibitors of cellular proliferation.

## Introduction

The cell cycle is a tightly regulated process required to guarantee the correct transmission of genetic information to daughter cells. The basic mechanism governing progression through the cycle is mediated by CDK/cyclins [1]–[4], a basic mechanism conserved from lower to higher eukaryotes that also has additional sophisticated control checkpoints in mammalian cells [5]–[8]; although some CDK appear to be dispensable [9]. However the cycle occurs in many different cell types ranging from stem cells, transit amplifying cells or tumor cells; and also in the context of an extracellular environment that is conditioned by homo and heterotypic cell interactions as well as variable characteristics of the extracellular matrix [10]–[12]. These different environmental conditions suggest that additional pathways and proteins must impinge on cell cycle progression or regulation that may be dependent on either cell type or growth conditions.

Recently a new family of serine-threonine kinases has been identified in the human kinome. This family known as the vaccinia-related kinases is composed of three members. VRK1 is the most characterized member [13] and it phosphorylates several proteins such as p53 [14], c-Jun [15], ATF2 [16] among transcription factors, or Baf, a protein required for nuclear envelope assembly [17], [18]. VRK1 specifically phosphorylates p53 in Thr18 resulting in its stabilization and transcriptional activation [14], [19]; and the resulting accumulation of p53 induces a proteolytic downregulation of VRK1 protein levels forming a novel autoregulatory loop [20] that is disrupted in lung squamous cell carcinomas with p53 mutations [21]. It has been postulated that VRK1 plays a role in maintaining a p53 molecule in a readiness state so that it can respond to any minor damage before DNA replication occurs [19], this suggests that VRK1 must be necessary relatively early in the G1 phase of the cell cycle, probably before the restriction point. Several additional lines of evidence suggest that VRK1 might be playing a role in relation with cell cycle progression. In normal human squamous epithelium, VRK1 protein presents a pattern of expression that overlaps that of the Ki67 proliferation marker, and coincides with the transit amplification compartment [22]. A similar correlation with this marker has been also detected in human head and neck squamous cell carcinomas, where it was correlated with other markers related with the proliferation phenotype, such as CDK2, CDK6, cyclin A and B1, topoisomerase II and survivin [22]. The use of siRNA specific for VRK1 caused a defective cell division in a colon tumor cell line [19]. Furthermore, in *C. elegans* the inactivation of the unique Vrk-1 ortholog gene was embryonic lethal, and in adult worms presented a slow growth phenotype [23]. In murine hematopoietic embryo development there was an overexpression of VRK1 at the time of massive cellular expansion in days E11.5 to E13.5 [24], and a similar finding was observed in murine developing retina [25]. All these data are consistent with a role for human VRK1 in cell cycle.

In this work we have determined that human VRK1 is necessary for cell proliferation and thus play a role is some phase of the cell cycle. The *VRK1* gene is turned off immediately after serum withdrawal, although the protein is very stable and last for several days, and its expression is reactivated by addition of serum. The loss of VRK1 results in a block of cell cycle progression in G1/S. Expression of *VRK1* gene is similar to that of immediate early genes such as *MYC* or *FOS* and occurs before expression of *CCND1* (cyclin D1). VRK1 protein levels correlate with those of the PCNA antigen and are inversely correlated with those of inhibitors of the cell cycle, such as p27.

## Results

### *VRK1* gene expression and protein level are downregulated in serum-starved fibroblasts

**Figure 1:**
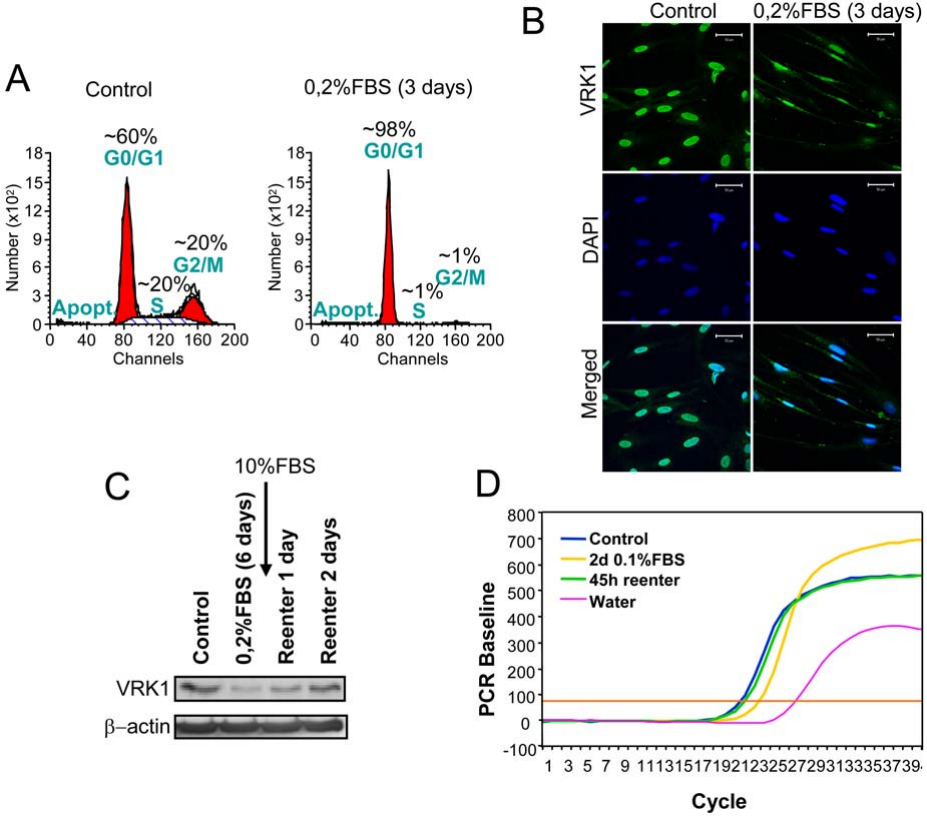
VRK1 levels in different growth conditions of human fibroblasts. (A). Flow cytometry profile of WS1 cell cycle in non-synchronized (left) and serum starved cells (right). (B). Nuclear localization of VRK1 and morphology of WS1 cells in non-synchronized and starved WS1 cells. Endogenous VRK1 protein was detected with a specific polyclonal antibody (VE1) in the cell nuclei. The size bar represents 50 µm. (C). Level of VRK1 endogenous protein determined in an immunoblot. (D). Change in VRK1 gene expression by qRT-PCR upon withdrawal or addition of serum to the cell culture. These experiments were independently performed three times.

The possibility that VRK1 protein level might be regulated by entry and exit in cell cycle was determined using the human normal fibroblast WS1 cell line. The cell cycle was stopped by serum withdrawal in the culture that was confirmed by flow cytometry analysis (Fig. 1A). These serum starved cells also underwent a phenotypic change becoming more elongated and with a lower intensity in endogenous VRK1 protein detectable by immunofluorescence (Fig. 1B). This observation was confirmed in an immunoblot, the serum withdrawal resulted in a drop in VRK1 protein level that was recovered upon serum readdition (Fig. 1C). This regulation appeared to take place at the transcriptional level, since the VRK1 message, determined by quantitative RT-PCR, dropped after serum withdrawal and was recovered after readdition of serum (Fig 1D). These results suggested that *VRK1* gene expression is regulated by exit and entry into the cell cycle at the transcriptional level and is regulated by serum.

### The expression of *VRK1* gene is regulated by serum at the transcriptional level

**Figure 2:**
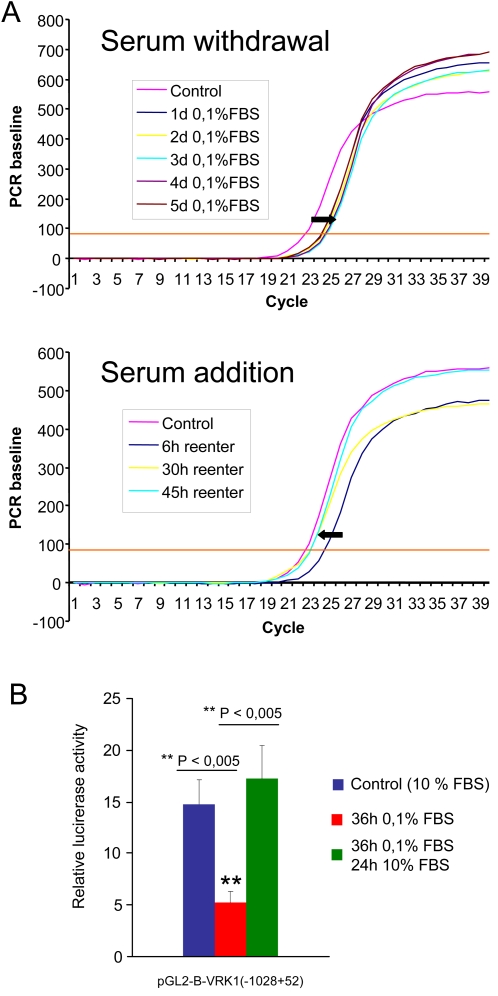
Changes in VRK1 RNA level upon serum withdrawal or readdition to the culture. (A). Quantification of the VRK1 gene expression in WS1 fibroblasts at different time points upon serum withdrawal (top) and serum readdition to the cell culture (bottom) which was previously serum-deprived for five days. These experiments were independently performed three times. (B). Regulation of the human proximal promoter of human VRK1 by serum. WS1 cells were transfected with plasmid pGL2-B-VRK1(−1028+52). The luciferase activity was determined under different experimental conditions. Control activity is that of cells in the continuous presence of serum. The result is the mean of three experiments.

It is known that the VRK1 protein is very stable, therefore it is actively downregulated by an active proteolytic process that is dependent on p53 [20], an inhibitor of cell cycle progression. However the previous data suggested that *VRK1* gene transcription can be regulated by signals that affected entry or exit into the cell cycle. Therefore it was determined by quantitative RT-PCR the change in specific VRK1 RNA levels upon exit and re-entry in cell cycle in response to serum at different time points. Upon serum withdrawal the VRK1 RNA level fell completely in one day (Figure 2A top), although the drop in protein level required several days. The re-entry in cell cycle by serum readdition also occurred within one day (Fig. 2A bottom). These data indicates that regulation of *VRK1* gene transcription is fast in response to the withdrawal, or readdition, of serum to the cell culture. Although the complete recovery of the protein level can take several days if the cells have been previously serum-deprived for several days. However, a second effect on VRK1 RNA stability can not be ruled out.

To confirm the effect of serum on *VRK1* gene transcription, the proximal promoter (−1028 to +52) of the human *VRK1* gene was cloned in a luciferase reporter construct. This construct was used to determine the effect of serum withdrawal, or readdition, on gene transcription measured by luciferase activity. The withdrawal of serum resulted in a loss of transcriptional activity, an activity that was regained by readdition of serum to the cell culture (Fig. 2B).

### *VRK1* is an early response gene in the G1 phase

**Figure 3:**
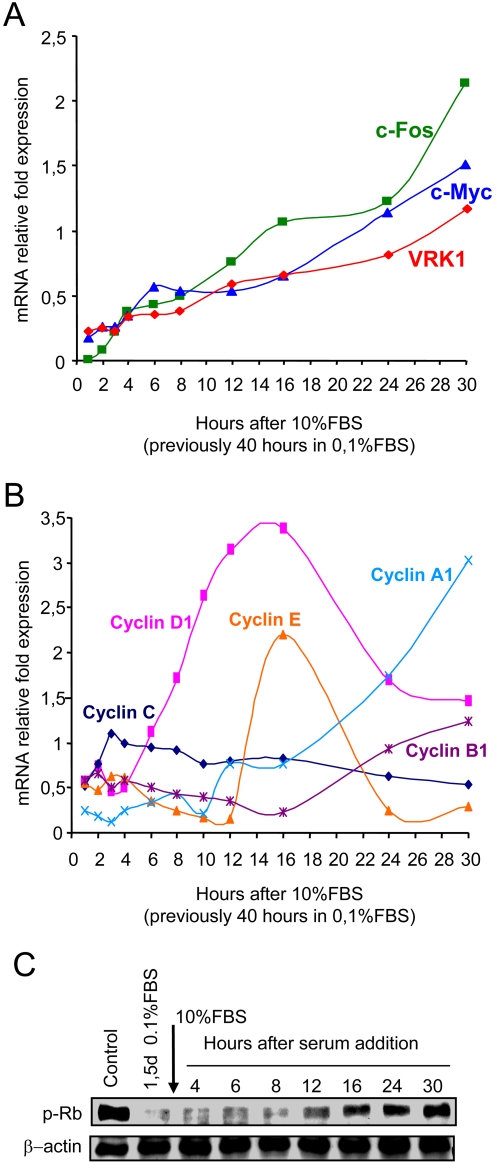
Cell cycle gene expression induced by removal or serum readdition. (A). Expression of *MYC*, *FOS* and *VRK1* genes after serum readdition to the culture. Before serum readdition cells were maintained for forty hours in 0.1% FBS. (B). Sequential expression of five cyclin genes after serum readdition to starved WS1 cells. (C). Timing of Rb phosphorylation after serum readdition to the starved WS1 cell culture detected with a specific anti p-Rb antibody. These experiments were independently performed three times.

The regulation of *VRK1* gene transcription by serum suggests that it might be regulated in the context of entry and exit in cell cycle progression. To determine if VRK1 is an early response gene, its expression after readdition of serum to starved cells was compared with that of two well established early response genes, such as *MYC* and *FOS* (Fig. 3A). The three genes, *VRK1*, *FOS* and *MYC* messages started to accumulate at approximately the same time, four hours after serum readdition. These data suggest that *VRK1*, like *MYC* and *FOS*, is also an early response gene.

Next it was determined at what point is VRK1 activated when entering the cell cycle in the WS1 fibroblast cell line. For this aim the timing of the temporal accumulation of VRK1 transcripts was correlated with the sequential expression of cyclin genes, since this is a cell line with a rather slow growth. Cyclin C, cyclin D1, cyclin E, cyclin A1 and cyclin B1 were consecutively expressed in the expected and sequential corresponding order with a start of their accumulation at 2, 6, 12, 16 and 17 hours respectively after serum readdition to starved cells (Fig. 3B). The best characterized cyclin D1 starts to be detected between four to six hours after serum readdition. The accumulation of VRK1, FOS and MYC RNA coincided, or started slightly sooner, than the time point of cyclin D1 accumulation, but these three RNA continue to accumulate, while those of cyclins peaked and drop sequentially in the correct sequential order within the G1 phase of the cell cycle. These results suggest that *VRK1* is an early response gene in cell cycle entry and is initially required for exit of G0 and in the early G1 phase.

Phosphorylation of Rb by the corresponding complexes of cyclinD1/cdk determines the progression of the cell cycle to enter in the G1/S transition [26]. The accumulation of phosho-Rb becomes clearly detectable after twelve hours (Fig. 3C), a time after VRK1 or cyclin D1 expression have already started to accumulate in this cell line.

### VRK1 protein level changes in relation to cell cycle related proteins

**Figure 4:**
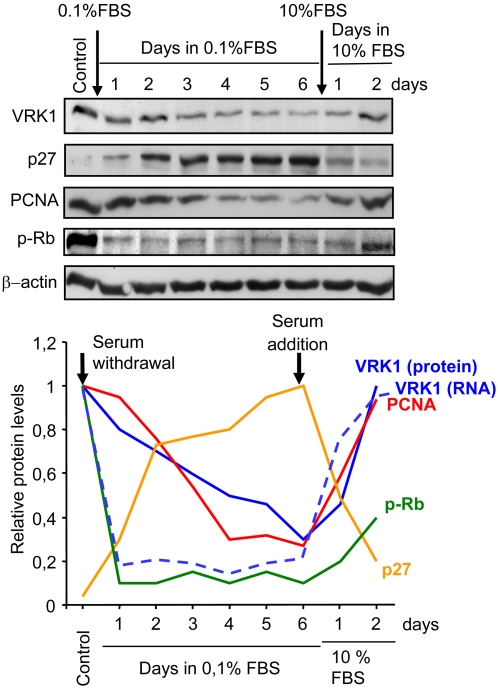
Time course of changes in cell cycle related proteins level after serum withdrawal and serum readdition to WS1 cells. The proteins were determined in an immunoblot with specific antibodies for each protein, VRK1, p27, PCNA and phospho-Rb. The level of actin was used as a loading control. At the bottom is shown the quantification of the level of each protein. In the case of VRK1 it is also shown the quantification by RT-PCR of its RNA (dashed line) at the same time points. These experiments were independently performed three times.

**Figure 5:**
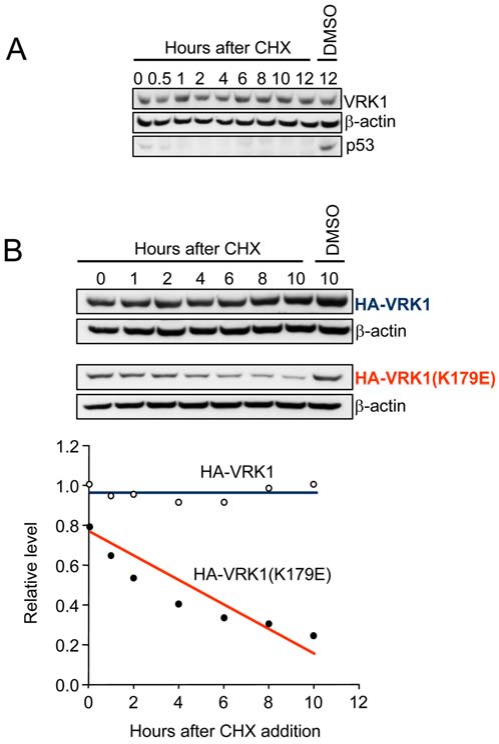
Stability of endogenous or transfected VRK1 protein. (A) The variation in levels of endogenous VRK1 and p53 proteins in HeLa cells was followed at different time points after addition of cycloheximide. The p53 protein drops in less than 30 minutes as expected, but there is no detectable change in VRK1 protein even after twelve hours suggesting it has a very high stability. (B). HEK293T cells were transfected with plasmids expressing wild-type human VRK1 or its kinase-dead K179E mutant. The stability of both proteins was followed at different time points after cycloheximide addition. Cells were transfected with plasmids pCEFL-HA-VRK1 or pCEFL-HA-VRK1(K179E) as previously described (Vega et al., 2004). These experiments were independently performed three times.

Next it was determined in WS1 fibroblast how the changes induced by serum deprivation in VRK1 protein levels behaved in relation to the levels of other proteins that also change during cell cycle progression. The PCNA protein, a DNA polymerase subunit required for DNA synthesis [27], as well as phosphorylated retinoblastoma (p-Rb) were used as markers of cell cycle activity [26], [28], [29]. The level of p27 was used as an indicator of cell cycle progression [30], [31]. The WS1 cells were starved by serum withdrawal for several days and the corresponding protein levels were determined each day. VRK1 levels dropped in parallel to those of phospho-Rb (p-Rb) or PCNA, which were reduced as a consequence of stopping the cell cycle. This reduction in levels was accompanied with the corresponding increase in the level of the cell cycle inhibitor p27 (Fig. 4) as expected if cell cycle progression is blocked [32], [33]. The readdition of serum induced a recovery of Rb phosphorylation, increased levels of VRK1 and PCNA, and reduction of the p27 inhibitor (Fig. 4). The fast drop in VRK1 RNA levels (dashed blue line) is not accompanied by a similarly fast drop in protein level, suggesting that the VRK1 protein (solid blue line) is very stable, which indicates that its half-life is approximately 4 days. This protein stability was confirmed by also determining the stability of transfected human VRK1, both active and kinase-dead in the presence of cycloheximide. The active kinase, endogenous or transfected is very stable, with a half-life of 4 days; while the kinase-dead VRK1 is much more unstable and has a shorter half life of a few hours (Fig. 5).

### siVRK1 induces a stop in the G1 phase of the cell cycle

**Figure 6:**
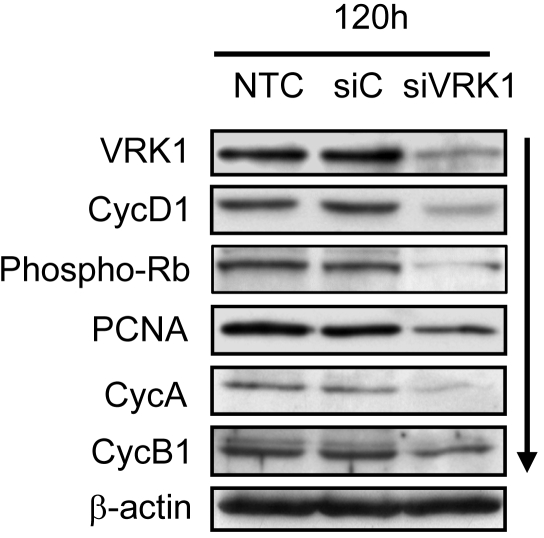
Effect of siVRK1 on proteins related with cell cycle progression. Levels of proteins in non-transfected, transfected with siRNA control, or with siRNA specific for VRK1 were determined in after 120 hours post transfection. The proteins were determined with the corresponding antibody indicated in the methods section, and identified in the corresponding immunoblot. These experiments were independently performed five times.

Previous data indicated that VRK1 might be necessary for cell cycle progression; therefore, its loss should be reflected as a loss of progression markers of the cell cycle. To established this possibility, it was determined if the loss of VRK1 induced by siRNA could have an effect on cell cycle progression markers. For this aim WS1 cells were transfected with either siControl or siVRK1, and different proteins that sequentially change during the cell cycle were also determined by immunoblot analysis. The loss of VRK1 was accompanied by a reduction of cyclinD1 and of p-Rb (Fig. 6), which indicates that these cells did not pass the restriction point in the G1 phase of the cell cycle. This observation is also supported by detection of a reduction in levels of cyclin A and cyclin B1, as well as the proliferation marker PCNA, which should have increased if these cells were able to progress towards DNA synthesis. These changes in cell cycle markers induced by siRNA VRK1 are consistent with the induction of an early block in the G1 phase of the cell cycle; these results are also similar, although mechanistically different, to those induced by serum deprivation in this cell line, and therefore consistent with a role of VRK1 in contributing to regulation of cell cycle progression.

### Loss of VRK1 by siRNA interferes with cell proliferation

**Figure 7:**
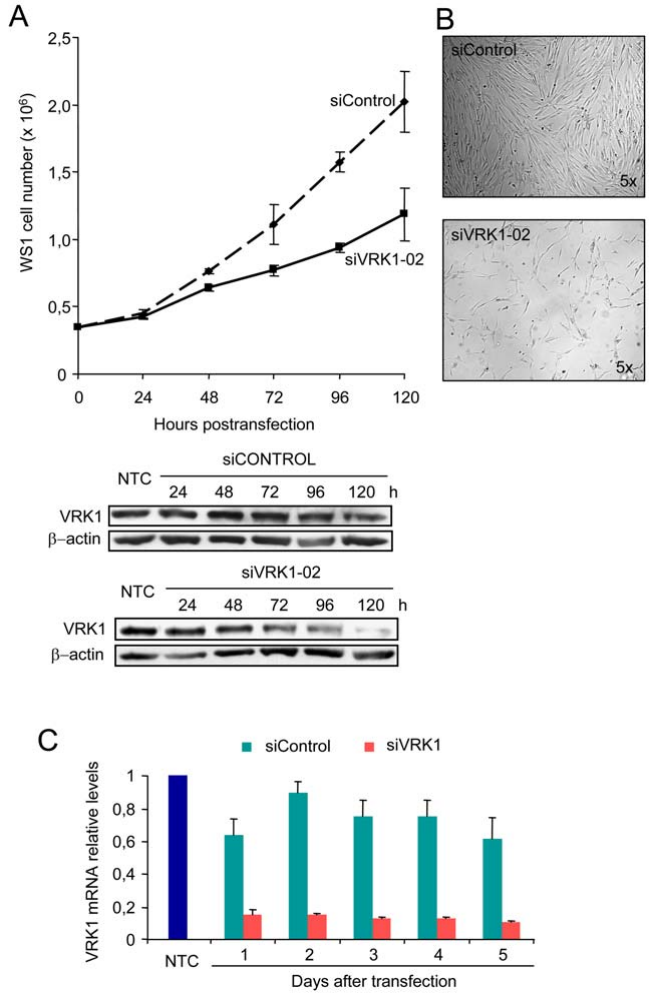
Effect of siRNA on endogenous VRK1 and cell proliferation. (A). Proliferation of WS1 cells that were transfected with siRNA control or siRNA specific for VRK1. The total cell number in the dish was determined at different time points (top). The endogenous protein was detected in an immunoblot (bottom) (B) Photograph of the WS1 cell cultures treated with siRNA control (top) or siVRK1 (bottom). The proliferation is much slower in cells transfected with the specific siVRK1. These experiments were independently performed three times. (C). Effect on VRK1 RNA levels in WS1 cells transfected with either siControl or siVRK1. NTC: non transfected cells.

The early expression of VRK1 in cell cycle entry suggests that it might be important for cell cycle progression. This observation is consistent with the data that associates VRK1 expression to the proliferation phenotype in tumor cell lines [34] and in human head and neck squamous cell carcinomas [22]. It was already known that in a human lung carcinoma cell line, loss of VRK1 by siRNA resulted in reduced proliferation [19]. Therefore, it was tested if in a cell line with a normal karyotype a similar effect was observed. To determine if the loss of VRK1 affects cell division of WS1 fibroblasts, these cells were transfected with siRNA control or siRNA specific for VRK1 and the progression of the culture was determined. The proliferation was slowed down as shown by a reduction in cell number when they were treated with siVRK1, but not in the control siRNA (Fig. 7A); a similar finding was detected when the total amount of protein was determined. After three days there was noticeable drop in endogenous VRK1 protein levels that was almost complete by day five, which was accompanied by an almost complete reduction in cell division as shown, both, by cell number or total mass. In the culture there were fewer cells after five days of specific siRNA treatment (Fig. 7B).

Also in these WS1 cells the effect of the siRNA control or specific siVRK1 was determined on the expression of the endogenous VRK1 message. The siVRK1 induced a reduction in the level of VRK1 RNA that reached its maximum effect the first day, and this low level was maintained throughout the time of observation (Fig. 7C).

### VRK1 and p63 in squamous epithelia

**Figure 8:**
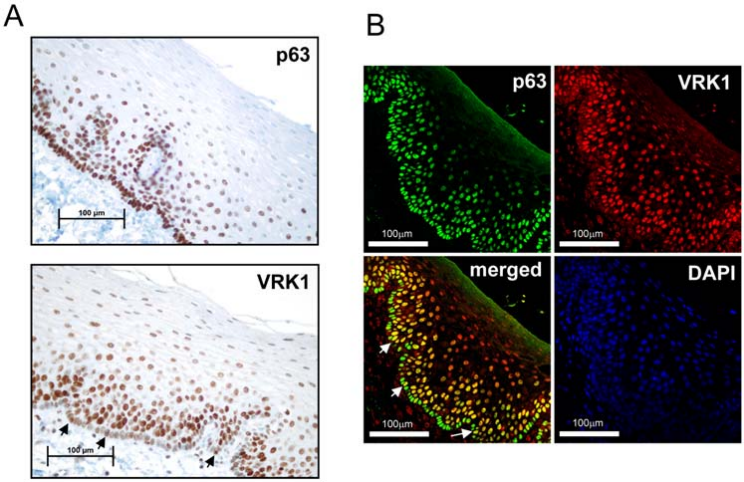
Localization of VRK1 and p63 in the squamous epithelium of the human pharynx. (A). Immunohistochemistry detection of p63 and VRK1 proteins in the pharynx. The arrows indicate cells (3 out of 56) in the basal layer with higher levels of VRK1. The p63 was detected with a monoclonal antibody. VRK1 was detected with the VC1 polyclonal antibody. (B). Immunofluorescence of p63 and VRK1 in the human pharynx epithelium. The arrows indicate cells (3 out of 57) in the basal layer that have higher levels of VRK1.

The p63 protein is mainly expressed in the basal layer of the epithelium and where there is cell proliferation, and some p63 isoforms has been associated with stem cells, this protein persist in the transit amplifying compartment [35]–[40]. Since VRK1 is induced by exit of G0, its expression should increase as a consequence of the first cell division. The expression of these two proteins, p63 and VRK1, was determined by immunohistochemistry in the human squamous epithelium of the pharynx. As expected the p63 signal strongly stains the first basal layer of cells and its intensity gradually decreases (Fig. 8A). VRK1 is weakly positive in the basal layer of the epithelium, although some individual cells present a stronger staining (arrow), and then suddenly increases reaching its highest intensity in the second layer of cells, consistent with the entry of these cells in the amplification compartment. Then VRK1 staining decreases till it disappears and differentiated cells become negative (Fig. 8A). The timing of the epithelial differentiation is in agreement with the half-life of VRK1 and its disappearance. The two proteins were also detected in immunofluorescence in pharynx (Fig. 8B), VRK1 overlaps with p63, but interestingly in the basal layer, the compartment where stem cells are located, only few cells have overlapping signal of VRK1 and p63, reflecting the higher level of VRK1 in these cells, and identifying a subpopulation within the basal layer of the epithelium that represents approximately five per cent of the cells in this compartment.

## Discussion

Regulation of cell cycle progression is a complex process in which the basic mechanism is determined by cyclins and cyclin-dependent kinases, and which is basically conserved from lower eukaryotes [1], [41]. However, in mammals, the cell is within a complex environment and its division can be affected by interactions with neighboring cells, both homotypic and heterotypic, the extracellular matrix as well as by the availability of growth factors; all of them sending signals that can control cell proliferation. This means that in mammalian cells there must be additional proteins implicated in cell cycle regulation that mediate its adaptation to its environment, and thus control tissue homeostasis.

In this context we have shown that VRK1 protein is necessary for cell division. VRK1 is likely to play different roles depending on the stage of cell cycle progression. It has to play a role in the exit from G0, since it is immediately expressed after readdition of serum to starved cells, and expression is turned on and off by serum. *VRK1* thus responds as an early response gene like *MYC* and *FOS*, two prototypical early serum-response genes. Once in the cell cycle is likely to contribute to activation of proteins needed for cell cycle progression, or its control, since this effect is mediated by phosphorylation of transcription factors, some of which have been identified, such as c-Jun, ATF2 or p53. Its requirement for cell cycle progression is supported by two observation, *VRK1* gene expression is inhibited as a downstream target of the p16INK4A-pRB-E2F pathway [42]; and overexpression of p16 in combination with non phosphorylated retinoblastoma also inhibit *VRK1* expression, since they block cell cycle progression [22]. In cells without retinoblastoma, that functionally mimic its phosphorylation, there is an overexpression of *VRK1* [43], consistent with the permanence of these cells in a cycling state.

But VRK1, which is a very stable protein, might have additional roles during cell cycle, as would be expected for a regulatory kinase. Thus in cells that are growing in a continuous way, as it occurs in cell culture o in the transit amplifying compartment of normal epithelium, where VRK1 levels are high and colocalize with the Ki67 proliferation markers [22]. In this situation, cells do not enter the G0 phase, VRK1 is necessary, since its loss will result in stoppage of cell division, as it occurs in fibroblasts (this work) or the HCT116 cell line [19] when they were treated with siRNA in the presence of serum. Among these roles for VRK1, there are two for which some evidence is already available. First VRK1 can contribute to maintain a level, albeit low, of p53 molecules in a readiness state so that it can respond immediately to suboptimal DNA damage in a dividing cell, without the need for induction of *TP53* gene expression, which requires hours. This action is mediated by the specific phosphorylation of p53 in Thr18 by VRK1 [14], [19], [44], [45]. In fact an autoregulatory mechanism between these two proteins has been identified, in this process VRK1 can not induce a permanent block of cell cycle progression by accumulation of a stabilized p53, because p53 induces proteolytic degradation of VRK1 [20], a mechanism that is disrupted in lung carcinomas with *TP53* mutations [21]; therefore in tumors with mutations in p53 there is an accumulation of a protein, VRK1, necessary for cell division, and thus might contribute to tumor cell expansion. This mechanism should be active before DNA replication takes place, thus it is necessary in the G1 phase of the cell cycle. Secondly, another moment in which VRK1 might be necessary during cell cycle progression occurs late in the cycle, just after chromosome segregation in mitosis, since VRK1 has been shown to regulate the Baf protein, which is necessary for assembly of the nuclear envelope [17], [18]; and it is also required for chromatin condensation, detected as phosphorylation of histone 3 [46]. In addition to these roles, it is likely that additional roles for VRK1 are identified in the future.

Thus we have reported that VRK1 protein is necessary for exit of the G0, and maintenance of cell cycle progression where it can play different roles depending on the phase of the cycle, and associated to its regulatory activity as a kinase. These roles will be defined by the future identification of specific VRK1 phosphorylation substrates in each phase of the cell cycle. The requirement of VRK1 for cell division indicates that it might be a suitable target for development of specific inhibitors aiming to control cell proliferation, which might be of therapeutic use in oncology as an alternative to current and more toxic inhibitors of cell division.

## Materials and Methods

### Cell lines and tissue culture

The human fibroblast WS1 cell line (CRL-1502) was obtained from the ATCC (Manassas, VA). Cells were grown in DMEM with 10% fetal bovine serum and supplemented with antibiotics and glutamine.

### RT-PCR

WS1 cells were washed in ice-cold PBS. Total RNA was extracted using the “RNeasy extraction kit” from Quiagen (Hilden, Germany). RNA was analyzed and quantified using a Bioanalyzer 2100 nano-lab chip from Agilent Technologies (Germany). 100 ng of total RNA were used in a one-step reverse transcription real-time PCR amplification reaction using the “Quantitec SYBR Green RT-PCR kit” from Qiagen in an iCycler (BioRad, Hercules, CA). The reaction was analyzed with iCycler software (BioRad) and PCR products were resolved in a 1.5% agarose ethidium-bromide gel. The following forward (F) and reverse (R) primers were used for specific human message detection. Human c-myc (Myc-F: 5′-CCAGCAGCCTCCCGCGACGATG-3′; Myc-R: 5′-GAGGGGTCGATGCACTCTGAGG-3′), human VRK1 (VRK1-F: 5′-CCAACGAGCTGCAAAACC-3′; VRK1-R: 5′-TGTCATGTAGACCAGACCCCC-3′), human c-fos (Fos-F: 5′-ATGATGTTCTCGGGCTTCAACGCAGCAG-3′; Fos-R: 5′-AACCAATTCTTACTATGGCAAGCG-3′), human cyclin C (CycC-F: 5′-GCCACTGCTACGGTATATTTCAAGAGATTC-3′; CycC-R: 5′-CAGAAGTAGCAGCAGCAATCAATCTTG-3′), human cyclin D1 (CycD1-F: 5′-CTTCCTCTCCAAAATGCCAG-3′; CycD1-R: 5′-AGAGATGGAAGGGGGAAAGA-3′), human cyclin E (CycE-F: 5′-GCAGAAGGTCTCAGGTTATC-3′; CycE-R: 5′-GTGGCCTCCTTAACTTCAAG-3′), human cyclin A1 (CycA-F: 5′-ATGGCATTTGAGGATGTGTATGAA-3′; CycA-R: 5′-CTGTGTTGAAATCCAGCAGGAA-3′), human cyclin B1 (CycB1-F: 5′-TCCATTATTGATCGGTTCATGC-3′; CycB1-R: 5′-TCAGTCACAAAAGCAAAGTCACC-3′), and GAPDH amplification was used as internal control (GAPDH-F: 5′-GGTCTTACTCCTTGGAGGCCATGT-3′; GAPDH-R: 5′-ACCTAACTACATGGTTTACATGTT-3′).

### Antibodies and immunoblot analysis

For determination of protein levels, cell extracts were prepared by homogenization in lysis buffer (Tris-HCl 50 mM pH 8, 200 mM NaCl, 5 mM EDTA, 1%Triton X-100 and protease and phosphatase inhibitors), and 40 micrograms were loaded for each sample in a 10% PAGE. Proteins were transferred to an Immobilon-P membrane (Millipore), blocked in TBS-T buffer with 5% non-fat milk, and then treated with the indicated first specific antibody for one hour, after which after the membrane was rinsed for 5 times, 5 minutes each time, and treated with the corresponding secondary antibody. The signals were detected using a chemiluminescence ECL kit (GE Healthcare). The signals were quantified in the linear response range using an FX Personal Imager (BioRad, Hercules, CA).

VRK1 protein was detected with either the VE1 rabbit polyclonal antibody, or the 1F6 murine monoclonal antibody [47]. P27 was detected with polyclonal antibody C-19; cyclin A with polyclonal antibody H-432; cyclin B1 with polyclonal antibody H-433; cyclin D1 with polyclonal M-20, and PCNA with monoclonal antibody PC10, all from Santa Cruz. Phosphorylated Rb was detected with polyclonal anti-pRb (Ser807/811) from Cell Signaling. All the antibodies were used at a 1∶1000 dilution. β-actin was detected with monoclonal (Clone AC-15) from SIGMA (St. Louis, MO) at a 1∶5000 dilution. Human p63 was detected with monoclonal antibody 4A4 from Dako (Glostrup, Denmark).

### Immunofluorescence and confocal microscopy

WS1 cells were plated on 10-cm2 dishes (5×10^5^) containing 1-cm-diameter sterile glass coverslips; after the corresponding serum treatment the slides were transferred to a tissue culture test plate. The cells were washed three times with PBS and then fixed in 4% paraformaldehyde in PBS for 30 min at room temperature. After fixation the cells were permeabilized in cold PBS containing 0.2% Triton X-100 for 30 min and then treated with glycine 10 mM for 10 min at room temperature. The cells were blocked with a solution containing 1% bovine serum albumin in PBS, at room temperature for 30 min, and then incubated for 30 min at 37°C or 1 h at room temperature with a mix of anti-VRK1 (VE1) in blocking solution. The cells were washed three times with PBS 0.1% Triton X-100 and incubated with a goat anti-rabbit Cy2 labeled antibody (Amersham Biosciences) in blocking solution. The cells were washed three times with PBS 0.1%, Triton X-100 and stained with DAPI (4′, 6′-diamidino-2-phenylindol) (Sigma) 1∶1000 in PBS for 10 min at room temperature. The cells were washed with PBS, and slides were mounted with Gelvatol (Monsanto). Subcellular localization was analyzed with a Zeiss LSM 510 confocal microscope.

### Cloning of human *VRK1* gene promoter

The human *VRK1* gene promoter was cloned by PCR amplification using genomic DNA from a BAC containing this gene (RP11-187K17 BAC library). The proximal promoter selected for amplification comprised from position −1028 to +52. The primers used for amplification were: Forward: 5′-CCGGTACCTGAGGTCCTGAAGCTTACGC-3′ with a *Kpn*I site; and reverse: 5′-TAGGATCCGCCGACTCGTAACTCGGCAG-3′.wirh a *Bam*HI site. The amplified DNA was subcloned into the *Kpn*I-*Bgl*II restriction sites of vector pGL2-Luc (Promega). The plasmid generated in called pGL2-B-VRK1(−1028+52).The sequence of the cloned promoter was confirmed by DNA sequencing.

### Luciferase assay

WS1 cells were grown in Dulbecco's minimal essential medium (DMEM) supplemented with 10% fetal calf serum and antibiotics in 5% CO_2_ humidified atmosphere. For treat cells, three hours before transfection, the medium was changed to a 0.1% FBS DMEM. Cells were transfected with 0.5 µg of the pGL2-B-VRK1(−1028+52) and 0.5 µg of a *Renilla* luciferase vector (pRL-tk). After 36 hours cells were harvested or replaced with 10% FBS fresh medium and kept for another 24 hours. Luciferase assays were performed with the dual reporter luciferase assay kit (Promega). The experiment was performed three times, and each individual measurement was determined in triplicate assays.

### RNA interference experiments

Synthetic SMART selected siRNA duplexes were purchased from Dharmacon RNA Technologies (Lafayette, CO). The targeted sequence for VRK1 (Accession number NM_003384) was CAAGGAACCTGGTGTTGAA (duplex siVRK1-2) from Dharmacon. Functional siCONTROL non-targeting siRNA pool from Dharmacon was used as a negative control and fluorescently labeled siGLO Lamin A/C siRNA for silencing and transfection efficiency. Transfections of siRNA duplexes at 100–200 nM final concentration were carried out using Lipofectamine 2000 reagent (Invitrogen) in WS1 cells following manufacturer instructions. After transfection, cells were processed for western blot, immunofluorescence or video microscopy as indicated. Viable cells were identified and counted by exclusion of Trypan Blue staining. Also, the total amount of protein as a function of cell number was determined.

### Flow cytometry

Fibroblasts were washed three times in PBS and detached with 25% Trypsin-EDTA (Life Technologies-Gibco); detached cells were rinsed three times with PBS by centrifugation at 1500 rpm for 5 min in a bench centrifuge. The cell pellet was resuspended in 700 µl of 70% ethanol (precooled at −20°C) and kept at 4°C overnight. The cells were collected by centrifugation and resuspended in 500 µl of PBS with10 µl propidium iodide (stock at 1 mg/ml) and 5 µl of RNAase (stock at 50 mg/ml). The cell suspension was incubated for 1 hour at room temperature, after which it was pass by the flow cytometer. A minimum of 10,000 cells were used for analysis. Data acquisition was performed with the Cell Quest program (Becton-Dickinson) and analyzed with programs Paint-a-Gate and ModFit for the cell cycle profile (Becton-Dickinson).

### Immunohistochemistry and immunofluorescence in tissue sections

Biopsies from normal human tissues obtained with informed consent were fixed in formalin and embedded in paraffin. Sections (3 µm thick) were cut and transferred to positively (silanized) charged-surface glass slides. Sections were dried for 16 hours at 56°C followed by dewaxing and rehydration through a graded ethanol series and washed with PBS. To retrieve antigens the slides were treated in a pressure cooker for 2 minutes in 10 mM citrate buffer (pH6.5). Briefly, after incubation, immunodetection was performed with the DAKO EnVision Visualization Method (DAKO), with diaminobenzidine chromogen as the substrate [21]. Sections were counterstained with haematoxylin. VRK1 was detected with the VC1 polyclonal antibody [47] and p63 with monoclonal 4A4 (DAKO).

For immunofluorescence the tissue sections, after antigen retrieval, were blocked with 2% defatted-milk, and 0.1% Triton in PBS, followed by washing in PBS. The slides were sequentially stained with the antibodies, and between them they were washed with PBS. Slide were first stained with an anti-p63 murine monoclonal antibody for 1 hour, followed by α-mouse-FITC (1/400) for 40 minutes at room temperature, 40′ RT, next the VC1 (policlonal rabbit) (1/500) for 1 hour, and finally α-rabbit-Cy3 (1/200) (GE Healthcare) for 40 minutes. After staining the slides were incubated in the presence of DAPI to detect the nuclei. Cells were mounted with Vectashield (Vector Laboratories, Burlingame, CA) and used for examination in a Zeiss LSM510 confocal microscope.
